# High frequency of delayed milk delivery to neonates in tropical beef herds

**DOI:** 10.1111/rda.14188

**Published:** 2022-08-02

**Authors:** Jarud Muller, Luis Prada e Silva, Geoffry Fordyce

**Affiliations:** ^1^ Department of Agriculture and Fisheries Charters Towers Queensland Australia; ^2^ Queensland Alliance for Agriculture and Food Innovation The University of Queensland Gatton Queensland Australia; ^3^ Queensland Alliance for Agriculture and Food Innovation The University of Queensland Charters Towers Queensland Australia

**Keywords:** colostrum, globulin, growth, lactation, neonatal calves

## Abstract

Beef‐calf mortality rates across tropical and subtropical Australia are high, with sub‐optimal nutrition in pregnant cows being the primary risk. The nutritional deficiencies associated with calf mortality are the same as those associated with reduced milk yields. Although the highest mortality risk occurs during neonatal life, the role of inadequate milk delivery to beef neonates is not well established. This study investigated the frequency of low milk delivery in tropically adapted neonatal calves and the time for their dams to initiate full lactation in five management groups of Brahman and Droughtmaster calving cows in the dry tropics of northern Queensland, Australia. Change in calf weight in the days following birth was the primary measure of milk uptake. Plasma globulin concentration was used to indicate colostrum uptake. Across management groups, data were available on 250 calves for regression analysis of average daily gain vs. globulin and on 78 for plotting calf growth profiles. Calves had one of two growth profiles, either with immediate high growth from birth (day one) or with high growth delayed until day three. The frequency of delayed growth calves (with inadequate milk intake to gain at least 0.5 kg by day three after birth) was on average 30% across management groups, with management groups ranging 25%–50%. The frequency of calves growing ≤0.2 kg/day to day three was 15%–37%, depending on management group. The frequency of calves growing ≤0.2 kg/day to day five was 7%–20%, depending on management group. Calf globulin explained only 25% of the variation in calf average daily gain. Our study shows that a third of tropically adapted calves may experience a three‐day delay to initiation of full lactation by their dams. Although study conditions were relatively benign, any additional risks with milk delivery, such as those that occur widely in tropical and subtropical northern Australia, would place such calves at risk of dehydration and mortality. Calf plasma globulin should not be used as a standalone measure of adequacy of neonatal milk delivery, especially when comparing across herds. This study demonstrates a fundamental problem of high frequency in northern Australia. The underlying risks for delayed milk delivery should be considered in the quest for practical solutions to reduce tropically adapted beef‐calf mortalities.

## 1 INTRODUCTION

Calf wastage (reproductive loss between confirmed pregnancy and weaning) has significant impacts on production (Fordyce et al., 2021) and animal welfare (Mee et al., 2019; Mellor & Stafford, 2004). Calf wastage levels in tropical and subtropical Australian beef herds are typically 5%–15%, where levels exceeding 20% are not uncommon, and can be up to 40% (McCosker et al., 2020). Calf wastage is also prevalent in other tropical countries, with levels averaging 8%–48% for various regions of Indonesia (Talib et al., 2003) and 11%–23% for various regions of West Timor (Jelantik et al., 2008). The highest risk period for calf wastage is during neonatal life (the first week from birth), in both temperate herds (Bendali et al., 1999; Mõtus et al., 2018) and in tropical herds, where neonatal mortality accounts for two‐thirds of calf wastage (Bunter et al., 2013). Before the specific mechanisms explaining neonatal mortality may be investigated in tropical beef calves, the risk factors for overall calf wastage in commercial herds must be considered. Herds exposed to poor pre‐partum nutrition consistently have increased calf wastage (Fordyce et al., 2022). In addition, high ambient heat load around calving is associated with increased calf wastage within three of the four major land types in tropical and subtropical Australia (Fordyce et al., 2022). Both poor pre‐partum nutrition for cows and high ambient heat loads around calving are risks that occur frequently across tropical and subtropical Australian herds. A decline in pasture digestibility and protein content occurs during the dry season in northern Australia (McIvor, 1981; Norman, 1963; Robinson & Sageman, 1967; Squires & Siebert, 1983), which typically occurs from March through to November–January. Peak calving in northern Australia occurs around the end of dry season (Bortolussi et al., 2005), that is late spring–summer. Therefore, the progression of pregnancy in cows coincides with a decrease in the quality of diet selected from the pasture, and calving cows are exposed to increasing ambient heat loads. The reason for timing reproduction in this way is to optimize overall reproductive performance and production (Hamlyn‐Hill, 2011; McCosker et al., 2022).

While the primary risk factors for overall calf wastage provide useful context (Fordyce et al., 2022), the majority of neonatal mortalities in tropical beef herds remain unexplained (Bunter et al., 2013). The nutritional deficiencies associated with calf wastage are the same as those associated with reduced milk yields, especially deficiencies of energy (Banchero et al., 2015), protein (Cowan et al., 1980), phosphorus (Castells et al., 2014) and water (Murphy et al., 1983). While high ambient heat loads around calving are associated with increased calf wastage (Fordyce et al., 2022), they are also associated with reduced milk yields (Brody, 1956; West et al., 2003). If neonatal calves do not receive milk, they die within three days at comfortable ambient temperature or within one day under hot conditions (Fordyce et al., 2015). The key mechanism mediating the effects of nutritional deficiency and/or high heat loads on calf mortality in tropical and subtropical Australia may therefore be low milk delivery and dehydration within the first week of birth. A lack of colostrum delivery, and thus, failure of passive immunity transfer is a major contributor to calf morbidity and mortality in intensively managed beef and dairy herds (Pérez et al., 1998). The relationship between level of passive transfer and mortality is well established in dairy calves (e.g. Donovan et al., 1998; Ibrahim & Lemma, 2009; Tyler et al., 1998), and there are some studies on failure of passive transfer to beef neonates in temperate environments (Filteau et al., 2003; Todd et al., 2018). In these production systems, failure of passive transfer predisposes neonates to diseases including diarrhoea and respiratory disease, which consequently are the most common causes of mortality and morbidity in young dairy cattle (Windeyer et al., 2014). The risk factors for calf mortality and morbidity in intensive systems include high stocking rate of calving cows (Radostits & Acres, 1980; Sanderson & Dargatz, 2000), cool moist conditions where all major enteric pathogens can survive for weeks to months (Anon., 2005; Millemann, 2009), contaminated environments (Cho & Yoon, 2014) and poor drainage (Radostits & Acres, 1980; Schumann et al., 1990; Singh et al., 2019). Risk factors are vastly different in tropical, extensively managed production systems, which are characterized by far lower stocking rates on lower quality pasture, and often extreme ambient heat through the calving period. In addition, calves in intensive systems that experience diarrhoea typically experience hyponatraemic dehydration (Groutides et al., 1990), secondary to fluid and solute loss through diarrhoea. This differs to dehydration in healthy milk‐deprived neonates that experience hypernatraemic dehydration, that is progressive loss of fluids while solutes are retained at an increasing concentration (Fordyce et al., 2015).

Colostrum uptake may be assessable using plasma globulin concentration (Zanker et al., 2001) as calves are born practically agammaglobulinaemic (Cabral et al., 2012). Milk delivery to calves may also be quantifiable by their weight change, as accrual of solid and fluid as tissue is primarily a function of milk uptake (Bartle et al., 1982; Black, 1982; Montsma, 1960; Totusek et al., 1973), and milk‐deprived calves lose weight primarily through body water loss (Fordyce et al., 2015).

The timing and frequency of low milk delivery in suckling neonatal beef calves in tropical environments have, however, not been investigated. If low milk delivery occurs at significant frequency, there may be opportunities to increase calf survival rates by managing cows so they produce and deliver adequate colostrum and milk for neonates. This study investigated the following in neonatal calves of tropical beef cattle herds: (1) What is the highest risk period for low milk delivery as measured by low growth from birth? (2) At what frequency does low milk delivery occur? and (3) Is calf plasma globulin concentration a potential indicator measure of milk delivery?

## 2 MATERIALS AND METHODS

### 2.1 Ethics approval

The specific research sites and respective ethics approvals were Spyglass native pasture (SA2013/08/441), Spyglass pens and Fletcherview pens (SA 2018/05/638), Spyglass improved pasture one (SA2018/09/651) and Spyglass improved pasture two (SA 2019/08/702). All approvals were granted by the Queensland Department of Agriculture and Fisheries Animal Ethics Committee.

### 2.2 Site calving environments

The study was conducted in the northern forest region of the Australian dry tropics. Five management groups of beef cows were included, across four separate sites, at Spyglass Beef Research Facility (Department of Agriculture and Fisheries) and Fletcherview Research Station (James Cook University). The study sites are denoted as Spyglass native pasture (*SPY‐NP*), Spyglass pens (*SPY‐P*), Fletcherview pens (*FV‐P*) and Spyglass improved pasture (*SPY‐IP*). *SPY‐NP* was a 95 hectare paddock dominated by native pasture species such as native black spear grass [*Heteropogon contortus*], kangaroo grass [*Themeda triandra*] and wiregrass [*Aristida sp*.], in an open Eucalyptus woodland (Table 1). *SPY‐P* and *FV‐P* were uncovered, dry lot pens (103 m^2^ per cow), where cattle were provided access to shade, and *ad libitum* water and hay. *SPY‐IP* comprised of improved pasture paddocks of 100–300 hectares, each with one or two watering points. The predominant pasture species in the improved, mostly cleared, pasture were Buffel grass (*Cenchrus ciliaris*) and Wynn cassia (*Chamaecrista rotundifolia*). Average maximum and minimum ambient temperatures during the calving periods of each management group are reported in Table 1. Total rainfall during the calving periods of management groups *SPY‐P*, *FV‐P* and *SPY‐IP1* were respectively 60, 60 and 130 mm. No rainfall >1 mm fell during the calving period of management groups *SPY‐NP* and *SPY‐IP2*.

**TABLE 1:** Description of cattle management groups assessed for variation in milk delivery to neonatal calves

Management group	Site name, and decimal latitude/longitude	Environment	Calving period	Temp range	BCS ± *SD* at calving	Breed (*n*)	Cow age ± *SD* (range)
*SPG‐NP*	Spyglass	Native pasture 95 ha	Aug–Sep 2013	15–29	2.7 ± 0.45	Brah (20)	5.1 ± 0.9 (4–6)
	BRF						
*SPG‐P*	−19.34/145.79	Six pens	Oct–Nov 2018	20–34	3.0 ± 0.3	DM (84)	3 ± 0.0 (3–3)
*FV‐P*	Fletcherview RS −19.88/146.18	Three pens	Nov–Dec 2018	22–34	2.9 ± 0.21	Brah (45)	5.5 ± 2.5 (3–12)
*SPG‐IP1*	Spyglass	Improved pasture 100–300 ha paddocks	Nov–Dec 2018	21–34	3.0 ± 0.42	Brah (23) DM (27)	5.1 ± 2.5 (3–12)
	BRF						
*SPG‐IP2*	−19.51/145.68	Improved pasture 130 ha paddock	Sep–Dec 2019	19–35	2.5 ± 0.53	DM (131)	3 ± 0.0 (3–3)

*Note*: Management group acronyms denote the environment in which calving occurred: *SPY‐NP*: Native pasture at Spyglass Beef Research Facility, *SPY‐P*: Pens at Spyglass, *FV‐P*: Pens at Fletcherview Research Station, *SPY‐IP1*: Improved pasture 1 at Spyglass and *SPY‐IP2*: Improved pasture 2 at Spyglass. Within the breeds column, counts indicate actual number of cows in paddock during study, including those that had calves with inadequate data to be included in the study.

*Abbreviations*: BCS, Body Condition Score; Brah, Brahman; BRF, Beef Researsch Facility; Cow age, in years; DM, Droughtmaster; RS, Research Station; Temp range, Average min and max temperature during calving period (°C).

### 2.3 Animal management

Calving occurred mid‐dry season for *SPY‐NP* and late‐dry season for all other management groups (Table 1).

*SPY‐NP*. Cows grazed the study paddock in the weeks prior to and during calving and were allowed *ad libitum* access to a supplement with expected individual daily intake of approximately 120 g (comprising 87% CP, 3.6% Ca, 3.4% P, 2.9% S, 1.5% fibre, 0.04% Mg and 1.96 MJ ME/kg).

*SPY‐P* and *FV‐P*. As part of an experiment, cows grazed in a paddock (one paddock per site) throughout pregnancy until birth of the first calf whereafter all animals were transferred to pens (on 5 October 2018 for *SPY‐P* and 12 November 2018 for *FV‐P*). At *SPY‐P*, cows were stratified based on live weight into two replicates, with each replicate containing three pens of different nutritional treatments: (1) Low‐quality Rhodes grass (*Chloris gayana*) hay fed *ad libitum*; (2) Rhodes grass hay plus 1.0 kg/cow day of a protein supplement (as fed: DM 91%, 35.5% CP, 7.9 MJ ME/kg); or, (3) The Rhodes grass hay plus the protein supplement with 14 g/cow of an added yeast extract (NaturSafe®, Diamond V). At *FV‐P*, cows were allocated into pens in the same way, but to only one replicate of the three treatments. Supplemented cows received protein meal for an average of 14 days prior to calving.

At *SPY‐IP*, two separate management groups of cattle were studied, *SPY‐IP1* and *SPY‐IP2*, in different years (Table 1). For each *SPY‐IP* management group, pregnant cows grazed large paddocks of approximately 1400 hectares of native pasture (with the native pasture species previously described). One month prior to predicted calving, early‐ and then late‐calving cows were transferred to the study paddocks. *SPY‐IP1* supplementation targeted an *ad libitum* intake (~2 kg/cow.day) of a molasses supplement fortified with urea and cracked corn (as fed: 77% DM, 18% CP, 7 MJ ME/kg) when dry season pasture had reduced in quality, to avoid weight loss of cows. *SPY‐IP1* cows were also allowed ad libitum access to molasses‐urea lick blocks (comprising 86% CP, 7% Ca, 3.6% P, 1.4% S and 10% fibre). In contrast, *SPY‐IP2* cows were not supplemented while in the calving paddock.

### 2.4 Measurements

In all management groups, cows were assessed for body condition score (1–5, 1 = emaciated and 5 = obese) on the day their calf was first assessed.

*SPY‐NP* calves and their dams were mustered daily from the day of birth until day seven of neonatal life, by a horseman between approximately 6:30 am and noon to cattle yards adjacent to the paddock. On a daily basis, calves were weighed to the nearest 0.5 kg using an aluminium platform scale (Ruddweigh Gallagher) and ~ 6 ml blood was sampled from the jugular into heparinized vacutainers that were placed immediately in an ice‐water bath until centrifugation and plasma collection 1–4 h later. Plasma samples were frozen for subsequent assays.

*SG‐P* and *FV‐P* calves were weighed at least three times per week (Mon, Wed, Fri) from the day of birth, using a calf cradle and analogue hanging scales (Model KC‐08, Kain Chung Scale Factory). Blood was sampled at the first two weighings, with plasma extraction and storage performed as per *SPY‐NP* calves.

*SPY‐IP1 and SPY‐IP2* calves were caught in the paddock using a side‐by‐side all‐terrain vehicle on day one of neonatal life and weighed and blood‐sampled as described for *SPY‐NP* calves. Calves were weighed using a calf cradle and hanging scales, with analogue scales for *SPY‐IP1*, and digital scales for *SPY‐IP2* (respectively Model KC‐08, Kain Chung Scale Factory at *SPY‐IP1*; and Brisbane Hunting Supplies, at *SPY‐IP2*). *SPY‐IP1* calves were each assessed for their second weighing and blood sampling at one of three yardings through the calving period (20 November 2018, 27 November 2018, or 5 December 2018) and were removed from the study after their second assessment. *SPY‐IP2* calves were weighed and blood‐sampled a second time during a weekly yarding of cows and calves from the calving paddock.

#### 2.4.1 Plasma analyses

Total plasma protein and albumin were determined by photometric colour test using an Olympus analyser (AU400; Beckman Coulter Inc.). Globulin concentration was calculated as total protein minus albumin. Serum rather than plasma was collected from *SPY‐IP2* calves, and serum globulin concentration was considered to be comparable with plasma globulin concentration for the purposes of this study (as per Elsohaby et al., 2019b).

### 2.5 Data management and statistical analyses

Separate analyses were conducted to (1) class individual calves based on their neonatal growth profile, (2) determine the frequencies of calves within each growth profile class and (3) determine whether there is an association between neonatal average daily gain (kg/day) and circulating globulin concentration. The experimental unit in all analyses using Genstat (v19.1) and R (1.3.1058) was the individual calf. Calf age was quantified as day of neonatal life, with the first 24 h from birth being day one. Calves excluded from the dataset prior to all study analyses were those whose dams had large difficult‐to‐suckle teats (*n* = 2), were stillborn (*n* = 3), experienced dystocia (*n* = 2), were not born until after the experimental calving period (*n* = 22), had average daily gain (ADG) exceeding 2 kg/day (*n* = 2), were too old at first weighing (*n* = 2, first assessment of neonatal life respectively being at days four and six) and those that were very weak and unable to stand and suckle at birth (*n* = 1).

As data were available from five separate management groups of calving cows with varying frequencies of calf assessments, only calves assessed at frequent time points through neonatal life could be classed for growth profile: *SPY‐NP* (*n* = 14), *FV‐P* (*n* = 48) and *SPY‐P* (*n* = 16; Table 2). Within each of these three management groups, calf growth from birth was analysed using restricted maximum likelihood (REML) with an AR1 covariance structure. Fixed terms included growth profile class, day of neonatal life and their interaction. The random term was the interaction of calf and day of neonatal life. Pairwise tests between predicted means were conducted using Fisher’s protected LSD procedure. Growth from birth through neonatal life was plotted as the REML predicted growth profile class means. For each management group, the percentage of calves not achieving >0.5 kg growth between birth and day three (weight calculated by interpolation where required) of neonatal life was calculated using raw data points. Distributions of calf ADG from the day of birth were plotted for each day of neonatal life within each management group.

**TABLE 2:** Counts of available data for regression analysis, plotting growth profiles, and counts of cows exposed to different supplementation treatments

	Management group						TOTAL
	*SPY‐NP*	*SPY‐P*		*FV‐P*	*SPY‐IP1*	*SPY‐IP2*	
Regression (n calves with data for ADG and globulin)	19	62		38	44	87	250
Supplement during calving period		**Rep 1**	**Rep 2**	**Rep 3**			
No supplement		11	8	16			35
High protein		14	7	11			32
High protein + yeast		11	11	11			33
Urea‐based dry lick	19						19
Fortified molasses +							
Urea‐molasses based							
Lick‐block					44		44
No supplement						87	87
Growth profile	14	48		16			78
Immediate growth	7	36		8			51
Delayed growth and ≥0.5 kg growth by day three	3						3
Delayed growth and <0.5 kg growth by day three	4	12		8			24

*Note*: Management group acronyms denote the environment in which calving occurred: *SPY‐NP*: Native pasture at Spyglass Beef Research Facility, *SPY‐P*: Pens at Spyglass, *FV‐P*: Pens at Fletcherview Research Station, *SPY‐IP1*: Improved pasture 1 at Spyglass, and *SPY‐IP2*: Improved pasture 2 at Spyglass.

Abbreviation: Rep, replicate group.

Within each management group, growth profile classes were compared for their birth weight using two‐sided *t*‐tests. Within management groups including dams of varying age (*SPY‐NP* and *FV‐P*), calf growth profile classes were compared for age of their dams at calving using Mann–Whitney–Wilcoxon tests. To compare globulin between growth profile classes within management group *SPY‐NP*, predicted means of plasma globulin were generated using REML analyses with the fixed terms: growth profile class, day of neonatal life and their interaction. The comparisons of calf growth profile classes for globulin within management groups *SPY‐P* and *FV‐P* were completed using the same terms, but in anova analyses, given data for only days one and three were available.

To determine whether there was a relationship between calf ADG and globulin, calf ADG was analysed using grouped linear regression, with explanatory variables: calf average globulin, management group and their interaction. The regression assumption of normally distributed residuals was tested by using normal probability plots. For the regression analysis, globulin values of calves were averaged to provide one datapoint per calf. Across management groups, the total number of calves with data available for both ADG and plasma globulin was 250. Available datapoints per management group and supplementation treatment are reported in Table 2. For *SPY‐IP2*, the early‐ and late‐calving cows were considered as separate management groups in the regression, given there was adequate sample size to do so (*n* = 40 and 47, respectively). Due to confounding with management group, other variables were not investigated in the regression analysis, including breed, cow age, cow body condition at day of calving, and whether cows were supplemented prior to calving or not (1 or 0).

## 3 RESULTS

**FIGURE 1:**
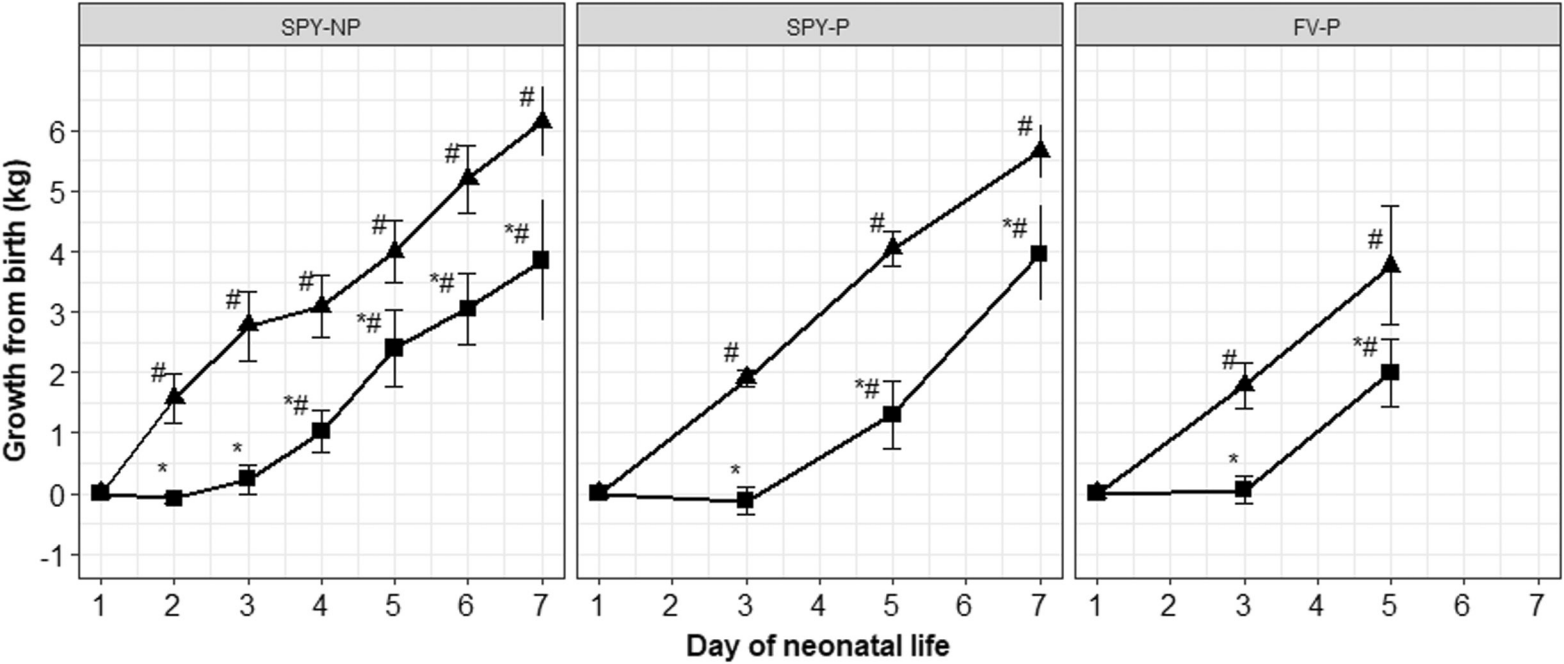
Growth from birth (± *SE*) for calves with immediate growth (▲) and delayed growth (■) from birth, using values predicted by restricted maximum likelihood (REML) analyses. Acronyms denote the environment in which calving occurred for each management group: *SPY‐NP*: Native pasture at Spyglass Beef Research Facility, *SPY‐P*: Pens at Spyglass, *FV‐P*: Pens at Fletcherview Research Station. * significant difference between growth profiles on a particular day within a calving environment; # significant growth from birth within a growth profile

It was found that calves had one of two classes of growth profile, immediate growth from the day of birth (day one) or with growth from birth delayed until day three (Figure 1). The frequency of calves with delayed growth (not achieving at least 0.5 kg by day three) was 29% for *SPY‐NP*, 25% for *SPY‐P* and 50% for *FV‐P*, and was 30.8% across calves in these three management groups. Delayed growth calves and immediate growth calves did not differ in birth weight for *SPY‐NP* (31.1 ± 2.9 vs. 27.9 ± 4.3 kg, *p* = .15); *SPY‐P* (30.4 ± 2.8 vs. 32.0 ± 3.8, *p* = .15); and *FV‐P* (30.7 ± 2.8 vs. 30.2 ± 5.3, *p* = .82). Dams of delayed growth and immediate growth calves did not differ for age at calving for *SPY‐NP* (5.1 ± 0.89 and 4.9 ± 0.9 years respectively, *p* = .59) and *FV‐P* (5.3 ± 1.9 and 5.9 ± 2.7 years respectively, *p* = .84).

**FIGURE 2:**
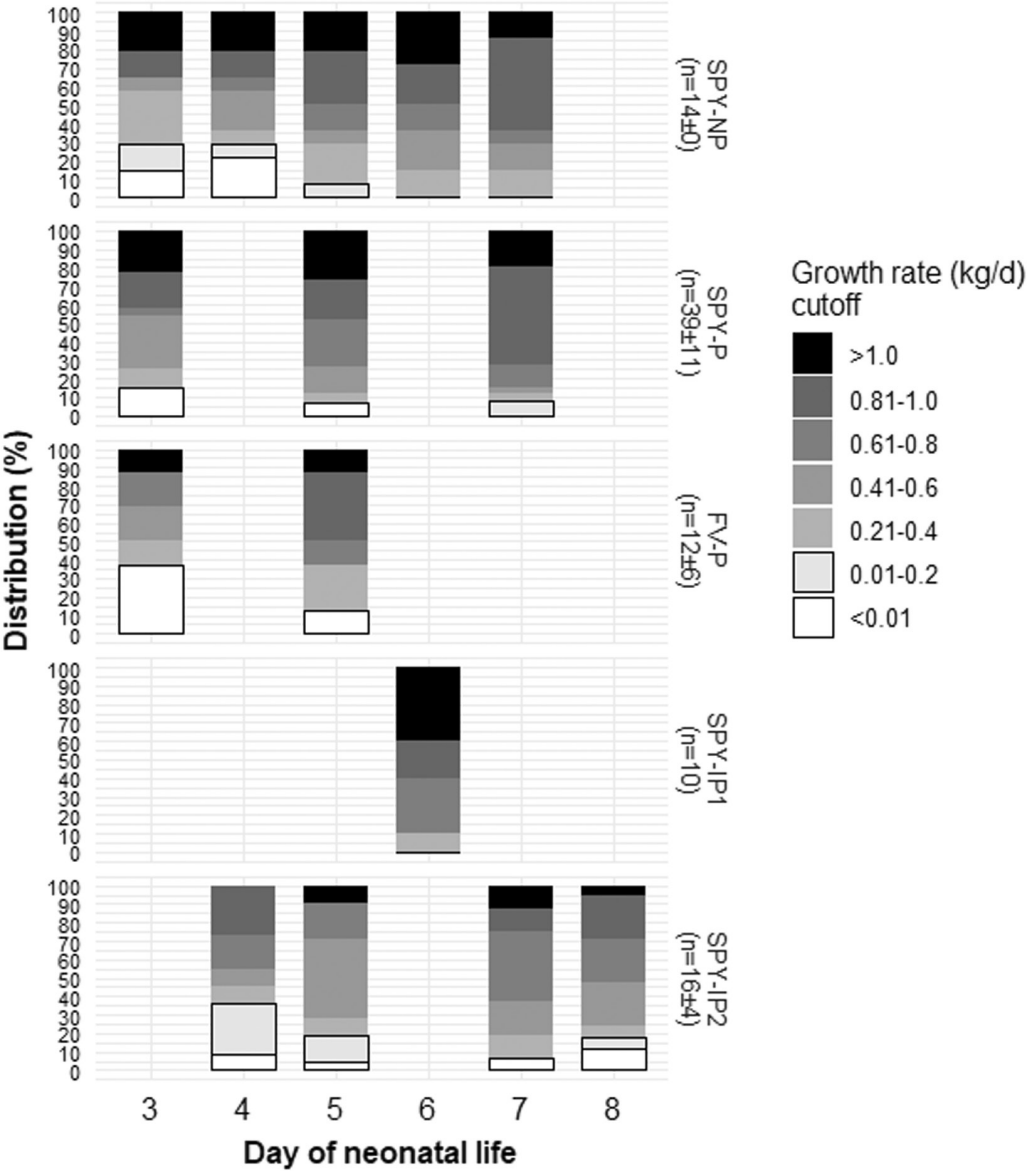
Distribution of average daily gain from the day of birth to various days of neonatal life within each management group. Acronyms denote the environment in which calving occurred for each management group: *SPY‐NP*: Native pasture at Spyglass Beef Research Facility, *SPY‐P*: Pens at Spyglass, *FV‐P*: Pens at Fletcherview Research Station, *SPY‐IP1*: Improved pasture 1 at Spyglass, and *SPY‐IP2*: Improved pasture 2 at Spyglass. In *SPY‐NP*, *SPY‐P* and *FV‐P*, the same set of calves were assessed for average daily gain at multiple points (days); while in *SPY‐IP1* and *SPY‐IP2* each calf was assessed for average daily gain once. Data are not shown where unavailable for particular days within a management group. Within a management group, the average ± *SD* number of datapoints across days is indicated by ‘*n*’

Figure 2 shows the distribution of calf ADG. The frequency of calves gaining ≤0.2 kg/day to days three and five of neonatal life were respectively 30% and 7% (*SPY‐NP*); 15% and 7% (*SPY‐P*); 37% and 13% (*FV‐P*). For *SPY‐IP1*, only day six data were available, where no calves grew ≤0.2 kg/day. For *SPY‐IP2*, no calves were assessed on day three and 20% of measured calves had ≤0.2 kg/day to day five.

*SPY‐NP* delayed growth and immediate growth calves differed significantly in plasma globulin at day one (38.9 vs. 56.9 g/L, *p* < .05), but not on subsequent days, that is growth profile class by day of neonatal life interacted in their effects (*p* = .01). Delayed growth and immediate growth calves did not differ in plasma globulin for *SPY‐P* (38.0 vs. 46.5 g/L, respectively; *p* = .08), or *FV‐P* (32.0 vs. 37.0 g/L; *p* = .55). Globulin and day of neonatal life were not associated for *SPY‐P* (*p* = .13) but were for *FV‐P* (*p* = .04). The interaction between growth profile and age was not significant for *SPY‐P* and *FV‐P* (*p* = .32 and .84, respectively).

Though there was no relationship between calf ADG and globulin when management group was not included in the regression model (*p* = .11), it was significant with management group included (*p* < .001; Table 3). The interaction between globulin and management group was not significant (*p* = .95).

**TABLE 3:** Grouped linear regression model to predict neonatal calf average daily gain, using calf average globulin concentration during neonatal life and management group

Parameter	Estimate	*SE*	*p* value
Calf average globulin concentration (g/L)	0.00423	0.00161	<.01
Management group			
*SPY‐NP*	0.707	0.0968	<.001
*SPY‐P*	0.819	0.0760	<.001
*FV‐P*	0.847	0.0860	<.001
*SPY‐IP1*	0.794	0.0832	<.001
*SPY‐IP2* (Early calving)	0.461	0.0868	<.001
*SPY‐IP2* (Late calving)	0.384	0.0840	<.001

*Note*: Regression adjusted *R* ^2^ = .25; overall regression *p* value < .001. Acronyms denote the environment in which calving occurred for each management group: *SPY‐NP*: Native pasture at Spyglass Beef Research Facility, *SPY‐P*: Pens at Spyglass Beef Research Facility, *FV‐P*: Pens at Fletcherview Research Station, *SPY‐IP1*: Improved pasture 1 at Spyglass, and *SPY‐IP2*: Improved pasture 2 at Spyglass.

## 4 DISCUSSION

In this study, a third of tropically adapted suckling neonates barely received adequate milk to grow during the first three days of neonatal life. Timely milk delivery during the first three days of neonatal life is critical for calf survival, as milk‐deprived tropically adapted calves die within three days, even under comfortable ambient temperatures (Fordyce et al., 2015). Delayed milk‐delivery calves are hypothesized to be at higher risk of dehydration and mortality where additional risk factors for low milk delivery exist. Compared with cows in the current study, risk levels for inadequate milk delivery to neonatal calves are expected to be much higher in many commercial herds in northern Australia, particularly where nutrition is limited due to poor quality and/or inadequate forage. Many commercial paddocks are much larger than the study paddocks, with waters far apart, placing additional stress on cows. Although low colostrum intake by calves may explain a significant proportion of the calf mortalities occurring in northern Australia (Fordyce et al., 2022), the link between milk delivery within the first three days of birth, that is the period of colostrum delivery (Crowther et al., 1916), and risk of calf mortality has not been established in tropical beef calves, as it has in dairy calves (Donovan et al., 1998) and piglets (Quesnel et al., 2012).

The risk of delayed growth in calves occurred in both Brahman and Droughtmaster herds, and across different years and sites. The reoccurrence of this risk supports the hypothesis that an underlying fundamental mechanism is driving the delay. The environmental risks and biological mechanisms explaining the differential timing of milk delivery between neonates are unclear. Although the variation in timing and volume of milk delivery to beef neonates is poorly understood, it may be explained by variation in calf ability to stand and suckle (Kim et al., 1988) and/or variation in peri‐partum milk yields. As dams of both immediate‐ and delayed‐milk delivery calves were of the same age, cow age does not explain the difference. In the current study, there was a very low frequency of weak calves (data not shown), precluding investigation of effects of this risk factor on milk delivery. Cows would be at increased risk of delivering weak calves if on a low protein diet pre‐partum (Bull et al., 1974) where weak calves have delayed suckling after birth (Kim et al., 1988). While it appears that milk yields may be a primary contributor to the high frequency of inadequate neonatal milk delivery in this study, subclinical effects on calves due to calving difficulty should not be discounted. Bianco et al. (2017) reported a high frequency of neonatal encephalopathy as an outcome of dystocia in hospitalized calves. Although dystocia has a low frequency in the breeds and environments of the present research, the frequency of prolonged labour and its consequences have never been investigated.

Delayed milk delivery has been reported in other species, including humans. In women, very limited milk yields have been reported in the first two days post‐partum, with rapid increase in production by days three–four post‐partum, where milk yields were measured by change in weight of infants (Neville et al., 1988; Saint et al., 1984). In dairy cows, there is limited secretion of milk components in pregnancy (lactogenesis I). Copious secretion of key milk components including lactose, and consequent osmotic accumulation of milk fluid (lactogenesis II; McCance et al., 1959), does not occur until the four‐day pre‐partum period in dairy cows (Hartmann, 1973). The research reported here supports the hypothesis that beef cows undergo the same timing of lactogenesis II and that problems with lactogenesis may explain a delay in milk delivery to neonates.

The effects of cow diet quality on milk yields are known for tropically adapted beef cows in full, established lactation (galactopoeisis; Fordyce et al., 1996). If lactogenesis II occurs with similar timing in beef cows, and similar nutritional effects on lactation occur during the days prior to calving, this could impact the amount of milk calves receive in neonatal life. However, the hypothesis that diet quality influences early lactation yields remains to be confirmed. This hypothesis would need testing in calving cows, given that early lactation yields would be influenced by factors not present while cows are in fully established lactation. These factors include the competing nutritional demands of the foetus, and the cow systemic hormonal and metabolic profiles that support foetal development and parturition. Frequencies of calves with low average daily gain from birth (≤0.2 kg/day) were high in the management group of cows not supplemented around calving (20% and 18%, to days five and eight of neonatal life, respectively). In contrast, negligible frequencies of calves with low average daily gain (≤0.2 kg/day) occured at that late stage of neonatal life in the other management groups; where cows were supplemented around calving. Cow nutrition around calving, and body condition score at calving may at least partially explain this, given that the non‐supplemented management group calved in approximately condition score 2.5 (1–5 scale), while other management groups were approximately in condition score 3 at calving. While this study was not designed to investigate the effects of nutrition or body condition score at calving on neonatal milk delivery, it demonstrates that these effects should be further investigated.

This study demonstrated calf change in live weight from birth as a viable measure for quantifying milk delivery to tropically adapted neonatal calves. This is consistent with other research where weight change has been shown to be an accurate measure of milk uptake in infants (Meier et al., 1990; Scanlon et al., 2002) and has been associated with milk uptake in dairy calves fed known amounts of milk (Huber et al., 1984; Khouri et al., 1968). ADG in young, pre‐weaned calves can be influenced by factors other than calf globulin, including cow body condition (McBryde et al., 2013; Winks et al., 1978), breed (Silveira et al., 2019), cow parity (Fordyce et al., 1993; Silveira et al., 2019), and management and environmental factors around the calving period including forage quantity/quality available (Fordyce et al., 1993), supplementation strategy (Short et al., 1996; Silva et al., 2022) and ambient heat load (Shivley et al., 2018). Though these animals, management and environmental factors can have large effects on overall milk yields, they can also have effects on colostrum globulin concentration (Shearer et al., 1992). Therefore, the effects of these factors on overall calf ADG may be at least partially explained by colostrum globulin concentration. While these factors were not considered in this study, including management group in the regression analysis corrected for some of their variation and increased the strength of association between ADG and globulin. Despite a link between calf circulating globulin concentration and neonatal growth, as has occurred in dairy calves (Elsohaby et al., 2019a), globulin was not an accurate predictor of neonatal ADG across different management groups. Therefore, a globulin threshold value was not identified, and globulin cannot be used as a stand‐alone measure to quantify milk delivery to neonates.

Further research is required to determine the specific risks associated with delayed or low milk delivery to tropically adapted beef neonates, especially that related to cow diet quality selected from pasture, supplementation strategy and ambient heat loads. If the use of neonatal calf live weight change as a measure of milk delivery, with cognisance of both quantity and quality of milk, can be further verified, it will increase the ease of calf survival research. Milk delivery to neonatal calves should be a primary consideration in studies of interventions such as the strategic use of high‐quality pastures and/or dam supplements for reducing calf mortality in tropical and subtropical regions.

## 5 CONCLUSION

Approximately a third of tropically adapted calves may experience a three‐day delay to initiation of full lactation by their dams. The risk of delayed milk delivery may place calves at higher risk of mortality under conditions that decrease potential milk production, increase the requirements of neonates for milk and/or reduce the capacity of the neonate to access milk. This risk appears to reduce after day four of neonatal life. Reduced colostrum delivery may also reduce immunocompetence and contribute further to risk of suckling‐calf mortality. Calf circulating globulin concentration is related to, though does not accurately predict calf growth and therefore overall milk delivery to neonatal calves. Consequently, a useful globulin threshold was not identified.

## AUTHOR CONTRIBUTIONS

Jarud Muller contributed to experimental design, project administration, managing and conducting field work, and writing. Luis Silva contributed to experimental design, project administration, supervision of project for management groups *SPY‐P* and *FV‐P,* and writing. Geoffry Fordyce contributed to experimental design, project administration, funding acquisition, overall supervision of all management groups, and writing.

## CONFLICT OF INTEREST

None of the authors have any conflict of interest to declare.

## Data Availability

The data that support the findings of this study are available from the corresponding author upon reasonable request.
